# Real-World Treatment Patterns and Clinical Outcomes After Platinum-Doublet Chemotherapy and Immunotherapy in Metastatic Non–Small Cell Lung Cancer: A Multiregional Chart Review in the United States, Europe, and Japan

**DOI:** 10.1200/GO.23.00483

**Published:** 2024-03-14

**Authors:** Ticiana A. Leal, Anandaroop Dasgupta, Dominick Latremouille-Viau, Carmine Rossi, Pragya Rai, Fabrice Barlesi, Stephen V. Liu

**Affiliations:** ^1^ Winship Cancer Institute, Emory University, Atlanta, GA; ^2^ Eisai Inc, Nutley, NJ; ^3^ Analysis Group, Inc, Montréal, QC, Canada; ^4^ Merck & Co., Inc, Rahway, NJ; ^5^ Paris Saclay University & Gustave Roussy Institute, Paris, France; ^6^ Lombardi Comprehensive Cancer Center, Georgetown University, Washington, DC

## Abstract

**PURPOSE:**

To characterize treatment patterns and real-world clinical outcomes of patients with metastatic non–small cell lung cancer (mNSCLC) who developed progression on an anti–PD-1/anti–PD-L1, herein referred to as anti–PD-(L)1, and platinum-doublet chemotherapy.

**METHODS:**

Eligible oncologists/pulmonologists in the United States, Europe (France, Germany, and United Kingdom), and Japan completed electronic case report forms for patients with mNSCLC (no evidence of *EGFR/ALK/ROS1* alterations). Eligible patients had disease progression on/after an anti–PD-(L)1 and platinum-doublet chemotherapy (received concurrently or sequentially), initiated a subsequent line of therapy (LOT) between 2017 and 2021, and had an Eastern Cooperative Oncology Group (ECOG) performance status 0-2 at this subsequent LOT initiation (index date). Overall survival (OS), time to treatment discontinuation (TTD), and real-world progression-free survival (rwPFS) after index were assessed using Kaplan-Meier analysis.

**RESULTS:**

Overall, 160 physicians (academic, 54.4%; community, 45.6%) provided deidentified data from 487 patient charts (United States, 141; Europe, 218; Japan, 128; at mNSCLC diagnosis: median age 66 years, 64.7% male, 81.3% nonsquamous, 86.2% de novo mNSCLC; at line of interest initiation: 86.0% ECOG 0-1, 39.6% liver metastases, 18.9% brain metastases, 79.1% smoking history). The most common treatment regimens upon progression after anti–PD-(L)1/platinum-doublet chemotherapy were nonplatinum chemotherapy (50.5%), nonplatinum chemotherapy plus vascular endothelial growth factor receptor inhibitor (12.9%), and platinum-doublet chemotherapy (6.6%). Median OS was 8.8 months (squamous, 7.8 months; nonsquamous, 9.5 months). Median TTD was 4.3 months (squamous, 4.1 months; nonsquamous, 4.3 months). Median rwPFS was 5.1 months (squamous, 4.6 months; nonsquamous, 5.4 months).

**CONCLUSION:**

In this multiregional, real-world analysis of pooled patient chart data, patients with mNSCLC who had disease progression after anti–PD-(L)1/platinum-doublet chemotherapy had poor clinical outcomes with various treatment regimens, demonstrating an unmet clinical need for effective options after failure on anti–PD-(L)1 and platinum-doublet chemotherapy treatments.

## BACKGROUND

Among patients with metastatic non–small cell lung cancer (mNSCLC) with no actionable mutations, PD-1/PD-L1 blockade, with or without CTLA-4 inhibition, and platinum-doublet chemotherapy (either alone or in combination) are recommended as first-line (1L) treatment by the ASCO, European Society for Medical Oncology, and Japanese Lung Cancer Society guidelines.^1-3^ Despite initial responses to anti–PD-1/anti–PD-L1 (herein referred to as anti–PD-[L]1) with or without platinum-doublet chemotherapy, the majority of lung cancers will progress; intrinsic or acquired resistance is expected in most cases.^4-7^ In real-world studies of 1L pembrolizumab plus platinum-doublet chemotherapy, 34%-38% of patients discontinued treatment because of disease progression and 32%-39% required a subsequent line of therapy (LOT) over a median follow-up time of 22-31 months.^8,9^

**CONTEXT**

**Key Objective**
What are the real-world outcomes and treatment patterns of patients with metastatic non–small cell lung cancer (mNSCLC) who develop progression after treatment with both anti–PD-(L)1 and platinum-doublet chemotherapy?
**Knowledge Generated**
In one of the first analyses of clinical outcomes after previous exposure to both anti–PD-(L)1 and platinum-doublet chemotherapy, patients with mNSCLC in the United States, Europe, and Japan in this study largely received nonplatinum chemotherapy after progression. Real-world clinical outcomes were suboptimal, with a median overall survival of 8.8 months, time to treatment discontinuation of 4.3 months, and real-world progression-free survival of 5.1 months overall.
**Relevance**
These findings highlight the large unmet need for additional treatment options that may improve survival in later lines of therapy for patients with mNSCLC who have experienced progression on both anti–PD-(L)1 and platinum-doublet chemotherapy. Clinical trials of novel agents that may offer benefit in this patient population are needed.

There are limited effective treatment options and recommendations for later lines of therapy after progression on anti–PD-(L)1 and platinum-doublet chemotherapy.^1,2,10^ Treatment guidelines generally recommend chemotherapy, including docetaxel, for squamous/nonsquamous mNSCLC, or pemetrexed for nonsquamous mNSCLC (if not previously delivered).^1-3^ Addition of an antiangiogenic agent such as ramucirumab or nintedanib to docetaxel may be considered, depending on regional approvals and reimbursement policies, but evidence of the regimens' efficacy is largely inferred from clinical trials conducted before the introduction of anti–PD-(L)1.^11-13^ Other agents or combinations have been evaluated in recently completed clinical trials (eg, pembrolizumab plus ramucirumab in Lung-MAP; OSE2101 in ATALANTE-1) or ongoing clinical trials (eg, pembrolizumab plus ramucirumab in Pragmatica-Lung; datopotamab deruxtecan in TROPION-Lung01) of NSCLC after progression on an anti–PD-(L)1 and platinum-doublet chemotherapy.^14-17^

Studies have evaluated treatment patterns and real-world outcomes following progression after anti–PD-(L)1 or platinum-doublet chemotherapy,^18-23^ but there are limited analyses of patients who have progression after both. A few previously published studies reported median overall survival (OS) of 8.1-11.6 months among patients with advanced NSCLC who developed progression on 1L anti–PD-(L)1 plus platinum-doublet chemotherapy in clinical practice, but these studies included small sample sizes or focused on second-line (2L) chemotherapy regimens only.^24,25^ Preliminary reports of a number of US-based studies have described real-world treatment patterns and median OS (6.2-14.3 months) after progression on anti–PD-(L)1 and platinum-doublet chemotherapy using electronic health record data from the Flatiron database.^26-28^ There remains a need for additional analyses of treatment outcomes associated with various regimens among a global patient population. This retrospective, physician panel–based medical chart review study characterized treatment patterns and real-world outcomes of patients with mNSCLC who developed progression after an anti–PD-(L)1 and platinum-doublet chemotherapy in the United States, Europe, and Japan.

## METHODS

### Data Source

A noninterventional, physician panel–based medical chart review was undertaken in the United States, Europe (ie, Germany, France, and United Kingdom), and Japan. Eligible medical oncologists and/or pulmonologists were recruited from an existing physician panel in each country to complete a survey and collect data from eligible patient charts via an online case report form (CRF). Data were collected from June 06, 2022, to April 28, 2023, and were anonymized; no personal identifiable information were accessible to the study sponsor or researchers at any time. Therefore, the study received an exemption from the Pearl Independent Institutional Review Board.

### Inclusion Criteria

Physicians were eligible if they had (1) a medical oncology or pulmonology subspecialty, (2) experience treating adult patients with mNSCLC for ≥5 years, (3) treated ≥5 patients with squamous/nonsquamous mNSCLC in the past 5 years, including ≥2 patients in the past year, (4) experience using immunotherapy-based regimens for mNSCLC, and (5) direct access to charts of patients with mNSCLC at the time of CRF completion, including those of deceased patients or patients no longer under care.

**FIG A1:**
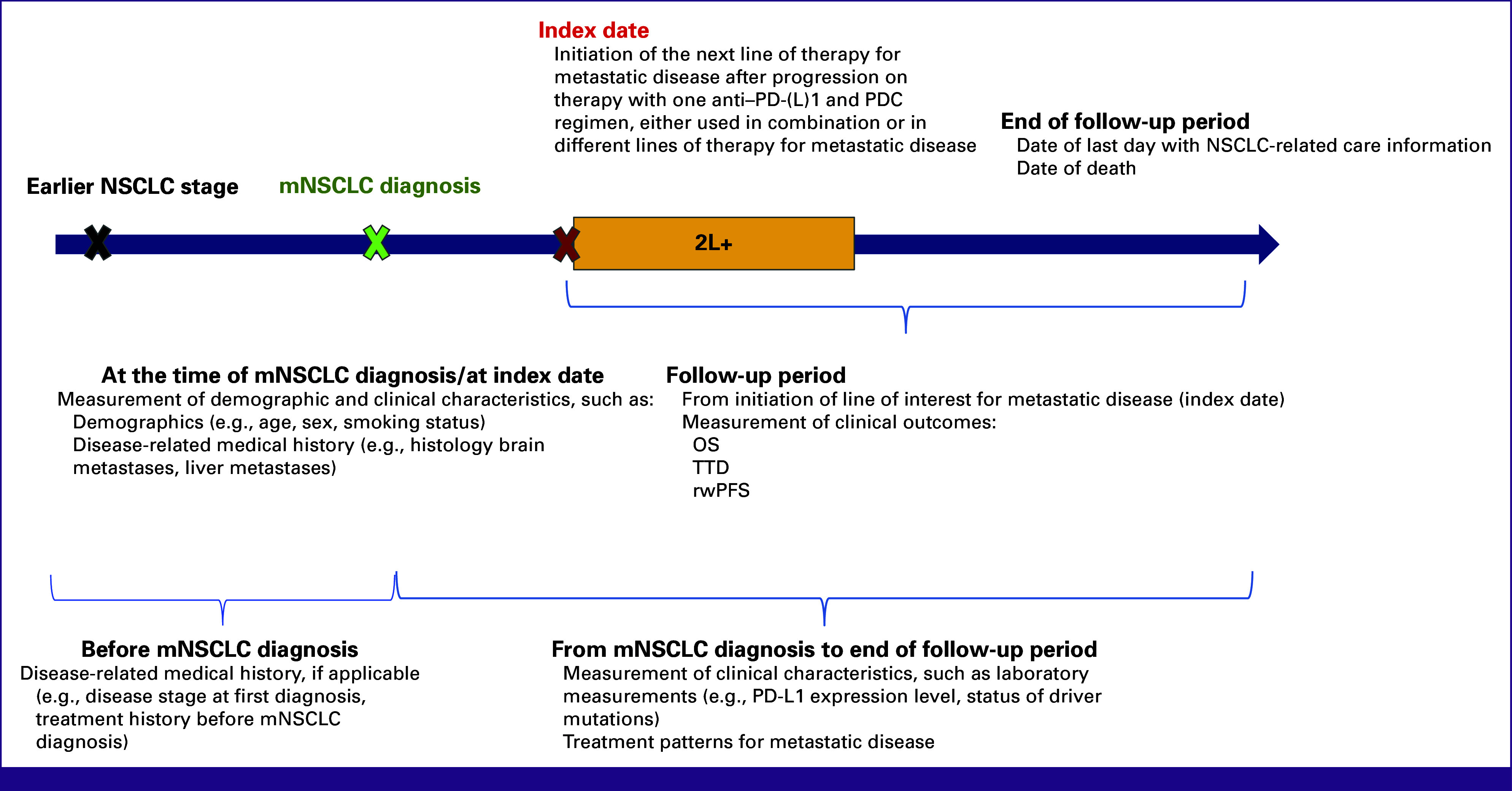
Study design scheme. 2L+, second-line or later; mNSCLC, metastatic non–small cell lung cancer; NSCLC, non–small cell lung cancer; OS, overall survival; PDC, platinum-doublet chemotherapy; rwPFS, real-world progression-free survival; TTD, time to treatment discontinuation.

Patient charts from eligible physicians were included if they (1) were from adults with a histologically or cytologically confirmed NSCLC diagnosis, (2) had mNSCLC with no history of other primary malignancies, (3) had not participated in a therapeutic clinical trial for mNSCLC, (4) had documentation of squamous (had no known *EGFR*-, *ALK*-, and *ROS1*-positive test results and no use of an *EGFR-*, *ALK-*, or *ROS1-*directed targeted therapy for mNSCLC) or nonsquamous (had documentation of *EGFR*- and *ALK*-negative test results and no known *ROS1*-positive test result) histology, (5) had progression after one anti–PD-(L)1 and platinum-doublet chemotherapy regimen (in combination or sequence for metastatic disease), (6) were initiated on a subsequent LOT (ie, 2L+ therapy; line of interest) between June 01, 2017 and September 30, 2021 (United States), or November 30, 2021 (Europe and Japan; given later availability of anti–PD-(L)1 in these regions), and (7) had an Eastern Cooperative Oncology Group (ECOG) performance status of 0-2 at this subsequent LOT initiation (ie, index date). Given patients with squamous histology are not commonly tested for *ALK* and *EGFR* mutations, negative test results were not required for eligibility. When physicians had multiple eligible patient charts, they were instructed in the CRF to select patients with last names beginning with programmed randomized letters. The study design scheme is presented in Appendix Figure A1.

### Measures and Study Outcomes

Physician characteristics, including biomarker testing patterns and prescription practices for mNSCLC (at the drug level), and patient demographic/clinical characteristics were assessed. The most common treatment regimens at the class level and treatment sequences for mNSCLC were reported by LOT, which included all treatments received for initial systemic and maintenance therapy. Temporary interruption or drop in treatment(s) of a regimen was not considered as a change in LOT. No minimum number of cycles was required to define a LOT. The identification of LOTs was based on physician assessment using these instructions included in the CRF.

OS was defined as the number of days from index date until death (any cause). Patients without death were censored at the end of the follow-up period, defined as the earliest of loss to follow-up or data collection time. Time to treatment discontinuation (TTD) was defined as the number of days from index date until treatment discontinuation (including death). Patients without discontinuation were censored at the end of follow-up. Real-world progression-free survival (rwPFS) was defined as the number of days from the index date until real-world progression or death (any cause), whichever occurred first. Patients who did not experience real-world progression were censored at the earliest of the initiation of a next LOT or end of follow-up. Real-world progression was defined using a clinician-anchored approach and was based on radiographic/imaging and/or physical examination/clinical evidence, as documented in the medical chart.

### Statistical Analyses

All analyses were descriptive, with no hypothesis testing performed. OS, TTD, and rwPFS were assessed using Kaplan-Meier analyses, with the median time to event along with 95% CIs reported if achieved. Subgroup analysis of end points was also conducted by histology type (ie, squamous and nonsquamous) and region (ie, United States, Europe, and Japan).

## RESULTS

### Physician Characteristics

Overall, 160 physicians (United States, 42; Europe, 71; Japan, 47) provided 487 patient charts (United States, 141; Europe, 218; Japan, 128). Most physicians were from academic-based practices (54.4%), treated >80 patients in the last year (43.8%), and had >10 years of experience treating patients with mNSCLC (82.5%; Appendix Table A1). Almost all physicians (99.5%) reported biomarker testing (next-generation sequencing [NGS], hotspot, or single gene) at initial mNSCLC diagnosis; physicians performed NGS testing for a mean of 85.2% of their patients. Physicians not testing patients at initial diagnosis reported histology (tissue/cell tumor type) among the reasons not to test at diagnosis (17.1%; higher in Europe [25.8%] than the United States [8.6%] or Japan [7.7%]), with fewer patients with squamous histology tested (mean of 48.2% of their patients with squamous histology). Approximately half (52.3%) of the physicians reported biomarker testing when tumor response was suboptimal/disease progression; these physicians performed NGS testing for a mean of 32.2% of their patients (18.9% in Japan).

### Patient Characteristics

Among the 487 included patients, median age was 66 years (IQR, 61-71), 64.7% were male, 86.2% were diagnosed with de novo mNSCLC, 18.7% had squamous histology, 86.0% had ECOG performance score 0-1, 39.6% had liver metastases, 18.9% had brain metastases, and 79.1% had a history of smoking (Table 1). PD-L1 expression level distribution was 27.1% with tumor proportion score (TPS) <1%, 49.5% with TPS 1%-49%, and 23.2% with TPS ≥50% (1 patient [0.2%] was not tested). The median theoretical and actual follow-up were 21 months (IQR, 17-30) and 8 months (IQR, 5-14), respectively. Most patients (n = 448, 92.0%) had progression after using anti–PD-(L)1 and platinum-doublet chemotherapy concurrently in 1L (ie, the line of interest for this study was 2L) and initiated the line of interest in 2021 (69.8%). Approximately 9.0% of patients had surgery for early-stage disease and 2.1% had radiation therapy in the neoadjuvant/postsurgery setting.

**TABLE 1:** Characteristics of Patients With mNSCLC Who Initiated a Subsequent Treatment After Disease Progression on Anti–PD-(L)1 and Platinum-Doublet Chemotherapy Received Concurrently or Sequentially for Metastatic Disease

Patient Characteristic	Pooled (N = 487)	By Histology		By Country		
		Squamous (n = 91)	Nonsquamous (n = 396)	United States (n = 141)	Europe (n = 218)	Japan (n = 128)
Age at mNSCLC diagnosis, years, median (IQR)	66 (61-71)	66 (60-71)	66 (61-71)	63 (57-68)	67 (62-72)	69 (64-72)
Male sex, No. (%)	315 (64.7)	65 (71.4)	250 (63.1)	89 (63.1)	142 (65.1)	84 (65.6)
Race/ethnicity,^a^ No. (%)						
White—Non-Hispanic/Latino				71 (50.4)		
Black or African American—Non-Hispanic/Latino				41 (29.1)		
Other				23 (16.3)		
Unknown				6 (4.3)		
De novo mNSCLC diagnosis, No. (%)	420 (86.2)	80 (87.9)	340 (85.9)	123 (87.2)	202 (92.7)	95 (74.2)
Squamous histology, No. (%)	91 (18.7)	91 (100.0)	0 (0.0)	11 (7.8)	59 (27.1)	21 (16.4)
Year of mNSCLC diagnosis, No. (%)						
≤2019	170 (34.9)	32 (35.2)	138 (34.8)	52 (36.9)	75 (34.4)	43 (33.6)
2020	196 (40.2)	35 (38.5)	161 (40.7)	57 (40.4)	97 (44.5)	42 (32.8)
2021	121 (24.8)	24 (26.4)	97 (24.5)	32 (22.7)	46 (21.1)	43 (33.6)
Index line of interest, No. (%)						
2L (anti–PD-(L)1+platinum-doublet chemotherapy in 1L)	448 (92.0)	85 (93.4)	363 (91.7)	127 (90.1)	209 (95.9)	112 (87.5)
3L+ (anti–PD-(L)1 and platinum-doublet chemotherapy sequentially from 1L-3L)	39 (8.0)	6 (6.6)	33 (8.3)	14 (9.9)	9 (4.1)	16 (12.5)
Year of line of interest initiation, No. (%)						
2017-2019	58 (11.9)	12 (13.2)	46 (11.6)	20 (14.2)	21 (9.6)	17 (13.3)
2020	89 (18.3)	17 (18.7)	72 (18.2)	25 (17.7)	40 (18.3)	24 (18.8)
2021	340 (69.8)	62 (68.1)	278 (70.2)	96 (68.1)	157 (72.0)	87 (68.0)
Duration of theoretical follow-up,^b^ months, median (IQR)	21 (17-30)	21 (16-30)	21 (17-30)	24 (20-33)	20 (16-28)	20 (16-33)
Duration of actual follow-up,^c^ months, median (IQR)	8 (5-14)	8 (5-12)	9 (5-15)	8 (5-16)	8 (5-13)	9 (5-14)
PD-L1 expression level, No. (%)						
Negative (<1%)	132 (27.1)	24 (26.4)	108 (27.3)	30 (21.3)	59 (27.1)	43 (33.6)
Intermediate (1%-49%)	241 (49.5)	46 (50.5)	195 (49.2)	77 (54.6)	112 (51.4)	52 (40.6)
High (≥50%)	113 (23.2)	21 (23.1)	92 (23.2)	34 (24.1)	47 (21.6)	32 (25.0)
Not tested	1 (0.2)	0 (0.0)	1 (0.3)	0 (0.0)	0 (0.0)	1 (0.8)
ECOG performance score at line of interest initiation, No. (%)						
0	89 (18.3)	12 (13.2)	77 (19.4)	18 (12.8)	27 (12.4)	44 (34.4)
1	330 (67.8)	65 (71.4)	265 (66.9)	97 (68.8)	152 (69.7)	81 (63.3)
2	68 (14.0)	14 (15.4)	54 (13.6)	26 (18.4)	39 (17.9)	3 (2.3)
Location of cancer metastases at line of interest initiation,^d^ No. (%)						
Brain	92 (18.9)	15 (16.5)	77 (19.4)	22 (15.6)	46 (21.1)	24 (18.8)
Stable brain metastases	43 (8.8)	5 (5.5)	38 (9.6)	7 (5.0)	17 (7.8)	19 (14.8)
Active brain metastases	51 (10.5)	10 (11.0)	41 (10.4)	16 (11.3)	29 (13.3)	6 (4.7)
Liver	193 (39.6)	37 (40.7)	156 (39.4)	67 (47.5)	94 (43.1)	32 (25.0)
Bone	223 (45.8)	39 (42.9)	184 (46.5)	52 (36.9)	118 (54.1)	53 (41.4)
Adrenal glands	135 (27.7)	22 (24.2)	113 (28.5)	33 (23.4)	75 (34.4)	27 (21.1)
Other (breast, kidney, skin)	82 (16.8)	14 (15.4)	68 (17.2)	8 (5.7)	43 (19.7)	31 (24.2)
Lymph nodes	265 (54.4)	40 (44.0)	225 (56.8)	85 (60.3)	121 (55.5)	59 (46.1)
Unknown	8 (1.6)	2 (2.2)	6 (1.5)	7 (5.0)	1 (0.5)	0 (0.0)
History of smoking before line of interest initiation, No. (%)	385 (79.1)	78 (85.7)	307 (77.5)	106 (75.2)	192 (88.1)	87 (68.0)

Abbreviations: 1L, first-line; 2L, second-line; 3L+, third-line or later; ECOG, Eastern Cooperative Oncology Group; mNSCLC, metastatic non–small cell lung cancer.

^a^ Race/ethnicity was only captured for patients in the United States.

^b^ Theoretical follow-up was defined as the period of time from the index date (ie, line of interest initiation) to the date the data were collected.

^c^ Actual follow-up was defined as the period of time from the index date (ie, line of interest initiation) to death or the earliest between the date the data were collected or the date of the last clinical visit, if patient remained alive.

^d^ Location of cancer metastases categories was not mutually exclusive.

Across regions, squamous histology was particularly lower in the United States, proportion of patients with ECOG score 2 was lower in Japan, and liver metastases were less common in Japan (Table 1). Race information was collected in the United States only; 29.1% were Black/African American.

### Treatment Patterns

**FIG 1:**
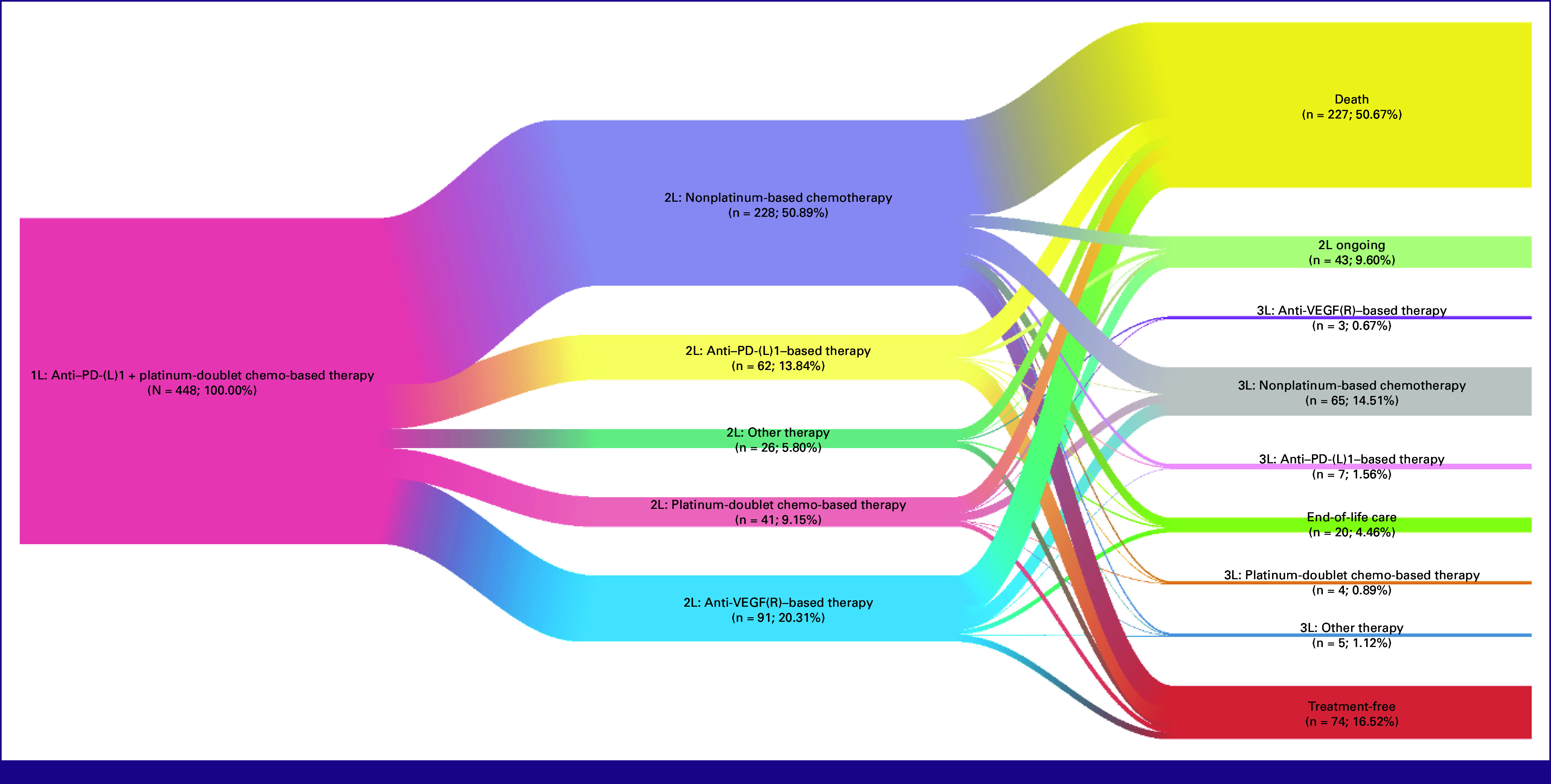
Treatment sequences of patients with mNSCLC who initiated a subsequent treatment after disease progression on 1L anti–PD-(L)1 plus platinum-doublet chemotherapy for metastatic disease. Anti–PD-(L)1–based therapy: 1L: anti–PD-(L)1 + platinum-doublet chemo, anti–PD-(L)1 + platinum-doublet chemo + (nonplatinum-based chemo/VEGF(R)/anti–CTLA-4); 2L: anti–PD-(L)1, anti–PD-(L)1 + (platinum-doublet chemotherapy/nonplatinum chemotherapy/anti–CTLA-4/BRAF). VEGF(R): VEGF(R) inhibitor, VEGF(R) inhibitor + (platinum-doublet chemo/nonplatinum chemo/anti–CTLA-4). Platinum-doublet chemo-based therapy: platinum-doublet chemo, platinum-doublet chemo + (nonplatinum chemo/anti–CTLA-4/targeted therapy [ie, BRAF, HER2, KRAS]). Nonplatinum-based chemotherapy: nonplatinum chemo, nonplatinum chemo + (anti–CTLA-4/target therapy [ie, BRAF]). Other: anti–CTLA-4, targeted therapy (ie, BRAF, EGFR, KRAS, MEK, MET, and RET). 1L, first-line; 2L, second-line; 3L, third-line; VEGF(R), vascular endothelial growth factor receptor.

The top five treatment regimens observed following progression after an anti–PD-(L)1 and platinum-doublet chemotherapy were nonplatinum chemotherapy (50.5%), nonplatinum chemotherapy plus vascular endothelial growth factor receptor (VEGF[R]) inhibitor (12.9%), platinum-doublet chemotherapy (6.6%), anti–PD-(L)1 monotherapy (6.2%), and platinum-doublet chemotherapy plus VEGF(R) inhibitor (4.7%; Table 2). Use of anti–PD-(L)1 was relatively more common in the United States. Among patients who discontinued the line of interest (89.3%), 20.5% initiated a subsequent LOT and 57.2% died before initiating a subsequent LOT. Among the 448 patients who developed progression after 1L anti–PD-(L)1 plus platinum-doublet chemotherapy, the most common treatment sequence was nonplatinum chemotherapy in 2L (50.9%) and nonplatinum chemotherapy in third-line (3L) therapy (14.5%; Fig 1).

**TABLE 2:** Treatment Regimens for the Subsequent Line of Therapy After Disease Progression on Anti–PD-(L)1 and Platinum-Doublet Chemotherapy Received Concurrently or Sequentially for Metastatic Disease

Treatment Characteristic	Pooled (N = 487)	By Histology		By Country		
		Squamous (n = 91)	Nonsquamous (n = 396)	United States (n = 141)	Europe (n = 218)	Japan (n = 128)
Most common treatment regimens observed during line of interest, No. (%)						
Nonplatinum chemotherapy	246 (50.5)	51 (56.0)	195 (49.2)	37 (26.2)	127 (58.3)	82 (64.1)
Nonplatinum chemotherapy + VEGF(R) inhibitor	63 (12.9)	9 (9.9)	54 (13.6)	10 (7.1)	32 (14.7)	21 (16.4)
Platinum-doublet chemotherapy	32 (6.6)	2 (2.2)	30 (7.6)	7 (5.0)	16 (7.3)	9 (7.0)
Anti–PD-(L)1	30 (6.2)	6 (6.6)	24 (6.1)	26 (18.4)	4 (1.8)	0 (0.0)
Platinum-doublet chemotherapy + VEGF(R) inhibitor	23 (4.7)	1 (1.1)	22 (5.6)	13 (9.2)	3 (1.4)	7 (5.5)
Anti–PD-(L)1 + PDC	21 (4.3)	6 (6.6)	15 (3.8)	9 (6.4)	11 (5.0)	1 (0.8)
Anti–CTLA-4	11 (2.3)	2 (2.2)	9 (2.3)	9 (6.4)	1 (0.5)	1 (0.8)
VEGF(R) inhibitor	9 (1.8)	1 (1.1)	8 (2.0)	4 (2.8)	2 (0.9)	3 (2.3)
BRAF inhibitor	7 (1.4)	0 (0.0)	7 (1.8)	4 (2.8)	3 (1.4)	0 (0.0)
KRAS inhibitor	6 (1.2)	0 (0.0)	6 (1.5)	4 (2.8)	1 (0.5)	1 (0.8)
Status at end of line of interest, No. (%)						
Ongoing treatment	52 (10.7)	7 (7.7)	45 (11.4)	16 (11.3)	23 (10.6)	13 (10.2)
Discontinued treatment	435 (89.3)	84 (92.3)	351 (88.6)	125 (88.7)	195 (89.4)	115 (89.8)
Remained treatment-free	75 (17.2)	10 (11.9)	65 (18.5)	49 (39.2)	22 (11.3)	4 (3.5)
End-of-life care	22 (5.1)	6 (7.1)	16 (4.6)	6 (4.8)	10 (5.1)	6 (5.2)
Died (may have received end-of-life care)	249 (57.2)	53 (63.1)	196 (55.8)	64 (51.2)	128 (65.6)	57 (49.6)
Initiated a subsequent line	89 (20.5)	15 (17.9)	74 (21.1)	6 (4.8)	35 (17.9)	48 (41.7)

Abbreviations: mNSCLC: metastatic non–small cell lung cancer; PDC, platinum-doublet chemotherapy; VEGF(R), vascular endothelial growth factor receptor.

For patients with squamous histology treated with nonplatinum chemotherapy regimen only, physicians responding to the survey (n = 61) reported mainly prescribing docetaxel (mean of 36.7% of their patients), paclitaxel/nab-paclitaxel (22.9%), and gemcitabine (15.4%); and for those with nonsquamous histology, docetaxel (mean of 34.2% of their patients), paclitaxel/nab-paclitaxel (20.2%), and pemetrexed (14.7%). For patients with squamous histology treated with nonplatinum chemotherapy plus VEGF(R) inhibitor regimens, physicians (n = 55) reported prescribing only docetaxel plus ramucirumab; and for those with nonsquamous histology (n = 60), mainly docetaxel plus ramucirumab (mean of 63.8% of their patients), docetaxel plus bevacizumab (20.5%), and docetaxel plus nintedanib (14.0%; 34.9% in Europe).

### Clinical Outcomes

**FIG 2:**
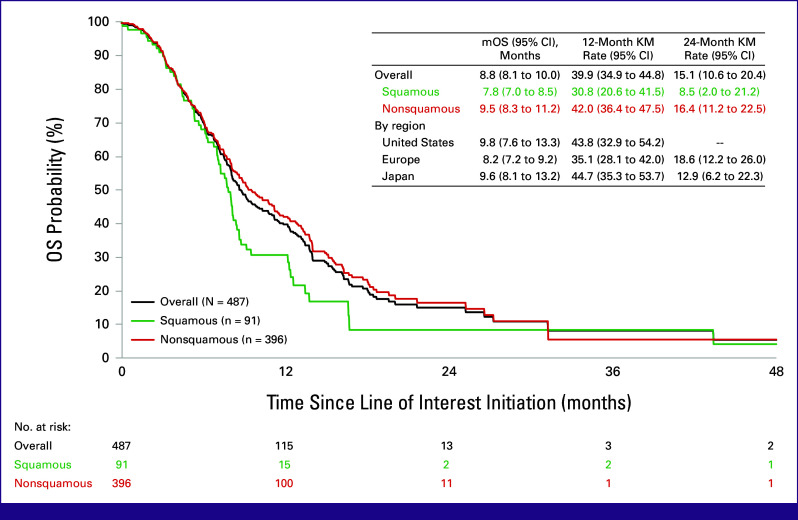
Overall survival of patients with mNSCLC who initiated a subsequent treatment after disease progression on anti–PD-(L)1 and platinum-doublet chemotherapy received concurrently or sequentially for metastatic disease. KM, Kaplan-Meier; mOS, median overall survival; OS, overall survival.

Overall median OS was 8.8 months (95% CI, 8.1 to 10.0), with 12- and 24-month OS rates of 39.9% (95% CI, 34.9 to 44.8) and 15.1% (95% CI, 10.6 to 20.4), respectively (Fig 2). For patients with squamous histology, median OS was 7.8 months (95% CI, 7.0 to 8.5), with 12- and 24-month OS rates of 30.8% (95% CI, 20.6 to 41.5) and 8.5% (95% CI, 2.0 to 21.2), respectively. For patients with nonsquamous histology, median OS was 9.5 months (95% CI, 8.3 to 11.2), with 12- and 24-month OS rates of 42.0% (95% CI, 36.4 to 47.5) and 16.4% (95% CI, 11.2 to 22.5), respectively. Median OS was 9.8 months (95% CI, 7.6 to 13.3), 8.2 months (95% CI, 7.2 to 9.2), and 9.6 months (95% CI, 8.1 to 13.2) in US, European, and Japanese patients, respectively.

**FIG 3:**
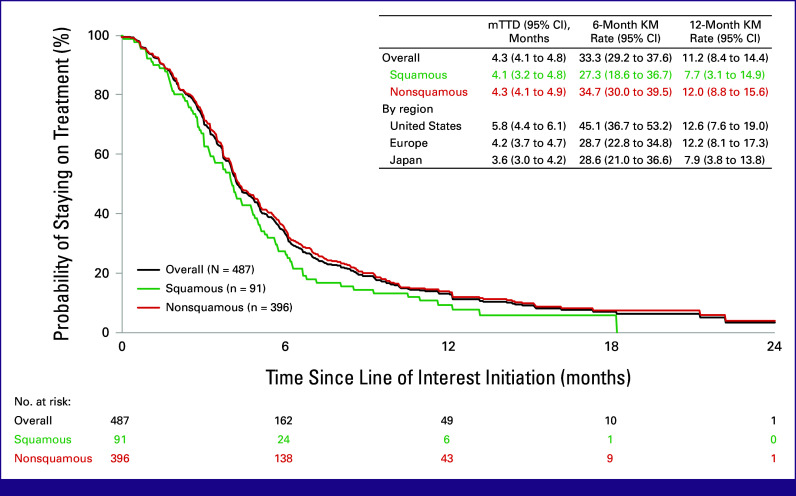
Time to treatment discontinuation of patients with mNSCLC who initiated a subsequent treatment after disease progression on anti–PD-(L)1 and platinum-doublet chemotherapy received concurrently or sequentially for metastatic disease. KM, Kaplan-Meier; mTTD, median time to treatment discontinuation.

Overall median TTD was 4.3 months (95% CI, 4.1 to 4.8), with 6- and 12-month on-treatment rates of 33.3% (95% CI, 29.2 to 37.6) and 11.2% (95% CI, 8.4 to 14.4), respectively (Fig 3). For patients with squamous and nonsquamous histology, the median TTD was 4.1 months (95% CI, 3.2 to 4.8) and 4.3 months (95% CI, 4.1 to 4.9), respectively. The median TTD was 5.8 months (95% CI, 4.4 to 6.1), 4.2 months (95% CI, 3.7 to 4.7), and 3.6 months (95% CI, 3.0 to 4.2) in US, European, and Japanese patients, respectively.

**FIG 4:**
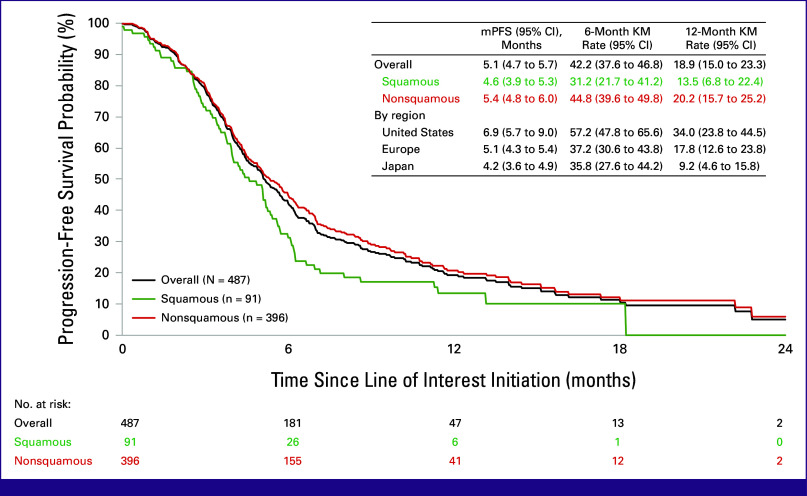
Real-world progression-free survival of patients with mNSCLC who initiated a subsequent treatment after disease progression on anti–PD-(L)1 and platinum-doublet chemotherapy received concurrently or sequentially for metastatic disease. KM, Kaplan-Meier; mPFS, median progression-free survival.

Overall median rwPFS was 5.1 months (95% CI, 4.7 to 5.7), with 6- and 12-month rwPFS rates of 42.2% (95% CI, 37.6 to 46.8) and 18.9% (95% CI, 15.0 to 23.3), respectively (Fig 4). For patients with squamous and nonsquamous histology, the median rwPFS was 4.6 months (95% CI, 3.9 to 5.3) and 5.4 months (95% CI, 4.8 to 6.0), respectively. The median rwPFS was 6.9 months (95% CI, 5.7 to 9.0), 5.1 months (95% CI, 4.3 to 5.4), and 4.2 months (95% CI, 3.6 to 4.9) in US, European, and Japanese patients, respectively.

## DISCUSSION

This contemporary study of patients with mNSCLC represents one of the first analyses of real-world clinical outcomes after previous exposure to both anti–PD-(L)1 and platinum-doublet chemotherapy, in combination or sequentially, among a large, pooled patient sample from the United States, Europe, and Japan. With majority of patients having PD-L1 expression <50%, most patients received anti–PD-(L)1 and platinum-doublet chemotherapy concurrently in 1L, consistent with guideline recommendations.^1-3^ Following progression after anti–PD-(L)1 and platinum-doublet chemotherapy, over half of the included patients received nonplatinum chemotherapy, with or without a VEGF(R) inhibitor, which was relatively consistent across histology and region. Real-world clinical outcomes were suboptimal, with a median OS of 8.8 months, TTD of 4.3 months, and rwPFS of 5.1 months overall, signaling an unmet need for more efficacious treatment options.

In this study, 50.5% of the patients received nonplatinum chemotherapy and 12.9% received nonplatinum chemotherapy plus VEGF(R) inhibitors after progression with an anti–PD-(L)1 and platinum-doublet chemotherapy. This observation is consistent with the multicenter study conducted by Auclin et al,^24^ where 46.0% of patients received taxane monotherapy and 20.1% received taxane plus antiangiogenic therapy in the 2L after progression on 1L immune checkpoint inhibitors plus platinum-doublet chemotherapy. Aside from the proportion of patients using nonplatinum chemotherapy in the current study, the remaining observed treatment patterns were quite heterogeneous, including retreatment with anti–PD-(L)1 agents and/or platinum-doublet chemotherapy. Few patients were observed to receive targeted treatment, because of the large intake of frontline genotyping in these centers (99.5% of physicians reported biomarker testing) and the study design excluding patients with common actionable mutations. Although reasons for treatment selection were not available in the data, it is possible that physicians tried regimens with no proven efficacy or guideline recommendation in the 2L+ setting because of the limited treatment options available. Indeed, Kundu et al^26^ observed similarly heterogeneous treatment patterns among patients with mNSCLC, with less than half receiving approved treatment options after progression on anti–PD-(L)1 agents and platinum-doublet chemotherapy.

The limited therapeutic options and use of nonrecommended regimens in 2L+ may have contributed to the poor clinical outcomes observed in this study. The overall median OS (8.8 months) was lower than what has been observed in the standard-of-care arms of recent clinical trials evaluating NSCLC after progression on anti–PD-(L)1 and platinum-doublet chemotherapy.^14,29,30^ For instance, median OS of the control arm was 11.6 months in Lung-MAP S1800A (72% received docetaxel with or without ramucirumab),^14^ 11.3 months in CANOPY-2 (docetaxel plus placebo arm),^30^ and 10.5 months in CONTACT-01 (docetaxel plus placebo arm).^29^ This difference may be due to the inclusion of patients with ECOG performance status of two (14.0%), and proportion of patients with brain (18.9%) or liver (39.6%) metastases in this study. Moreover, the definition of anti–PD-(L)1 resistance and extent of its use in early-stage disease may have varied across studies.

When comparing with previous real-world studies, the present median OS of 8.8 months was within range of the literature estimates of 6.2-11.6 months, while our median rwPFS of 5.1 months was slightly longer than the literature estimates of 2.9-4.1 months.^24-28^ However, variability in rwPFS can be affected by different disease monitoring practices (eg, frequency of follow-ups) and definitions of progression used in clinical practice, which may limit comparability between real-world studies.^31^ Patients in the Japanese REACTIVE study had the longest median OS (11.6 months), which was longer than that of the Japan subgroup in the current study (9.6 months), potentially because REACTIVE focused on the approved and guideline-recommended regimen of 2L docetaxel plus ramucirumab (only 12.9% of patients in the current study received nonplatinum chemotherapy plus VEGF[R] inhibitor).^2,25,32^ With regards to US studies, the median OS of 9.8 months among US patients in this study was longer than the estimates reported in previous preliminary analyses of Flatiron electronic health record data (range of 6.2-8.9 months).^26-28^ Although Auclin et al^24^ evaluated patients from several countries across North America and Europe, they did not report findings stratified by region. However, the authors did conduct a subgroup analysis by histology, with patients with adenocarcinoma having longer median OS and rwPFS than patients with squamous cell carcinoma. These results are consistent with the current subgroup analysis showing a trend of better clinical outcomes for patients with nonsquamous relative to squamous histology.

Although TTD is typically associated with PFS in clinical trials of mNSCLC,^33^ median TTD in the current study (4.3 months overall) was slightly shorter than rwPFS (5.1 months overall). This discrepancy may be explained by the aforementioned variability in disease progression monitoring in clinical practice^31^ or the fact that TTD was estimated on the basis of the last administration date of treatment, where progression would preclude a subsequent dose (and thus discontinuation would appear to occur earlier than disease progression). Additionally, since most patients initiated their line of interest in 2020-2021, the COVID-19 pandemic may have caused delays in patient follow-up to identify progression.^34^

Given the heterogeneous monitoring and treatment approaches in real-world clinical practice, these study findings may not be directly comparable with clinical trial results.

There were limited patients observed in 3L treatment or later after progression on anti–PD-(L)1 and platinum-doublet chemotherapy received sequentially from 1L-3L since most patients received 1L anti–PD-(L)1 plus platinum-doublet chemotherapy in combination. This may be because more than three quarters of the patients had PD-L1 expression levels with TPS <50%, for which combination therapy is typically recommended.^1-3^ Capture of sequential treatment with anti–PD-(L)1 and platinum-doublet chemotherapy may have been limited by early deaths or the available follow-up time, warranting further research in this 3L+ setting.

As with all chart reviews, the findings may have been influenced by selection bias, recall bias (eg, physicians may not have specifically remembered the reasons for including specific notes), and incomplete data on the basis of the omission of a particular answer option.

In conclusion, in this large, real-world analysis of pooled patient chart data from the United States, Europe, and Japan, patients with mNSCLC previously treated with anti–PD-(L)1 and platinum-doublet chemotherapy had heterogeneous treatment patterns after progression. This study contributes to the growing literature describing the poor clinical outcomes among patients with NSCLC who experience progression on anti–PD-(L)1 and platinum-doublet chemotherapy, highlighting the large unmet need for additional treatment options that may improve survival in later lines of therapy. Therefore, clinical trials of novel agents that may offer benefit in this patient population are needed.

## Data Availability

Data are not available because of legal restrictions. Because of the nature of this research, participants of this study did not agree for their data to be shared publicly, so supporting data are not available. Therefore, restrictions apply to the availability of these data, which are not publicly available.

## APPENDIX

**TABLE A1:** Physician Characteristics

Physician Characteristic	Pooled (N = 160)	By Country		
		United States (n = 42)	Europe (n = 71)	Japan (n = 47)
Primary practice setting, No. (%)				
Community-based	73 (45.6)	30 (71.4)	20 (28.2)	23 (48.9)
Academic-based	87 (54.4)	12 (28.6)	51 (71.8)	24 (51.1)
US census region, No. (%)				
Midwest		15 (35.7)		
South		12 (28.6)		
West		8 (19.0)		
Northeast		7 (16.7)		
Patients with mNSCLC treated in the last 12 months, No. (%)				
2-20	7 (4.4)	4 (9.5)	0 (0.0)	3 (6.4)
21-40	30 (18.8)	7 (16.7)	5 (7.0)	18 (38.3)
41-80	53 (33.1)	14 (33.3)	22 (31.0)	17 (36.2)
>80	70 (43.8)	17 (40.5)	44 (62.0)	9 (19.1)
>10 years of practice treating patients with mNSCLC, No. (%)	132 (82.5)	34 (81.0)	59 (83.1)	39 (83.0)
No. of contributed patient charts, median (IQR)	2 (1-5)	3 (1-5)	2 (1-4)	2 (1-4)

Abbreviation: mNSCLC: metastatic non–small cell lung cancer.
